# MicroRNA-9 regulates the expression of peroxisome proliferator-activated receptor δ in human monocytes during the inflammatory response

**DOI:** 10.3892/ijmm.2013.1311

**Published:** 2013-03-20

**Authors:** PETRA THULIN, TIANLING WEI, OLIVERA WERNGREN, LOUISA CHEUNG, RACHEL M. FISHER, DAN GRANDÉR, MARTIN CORCORAN, EWA EHRENBORG

**Affiliations:** 1 Atherosclerosis Research Unit, Department of Medicine, Center for Molecular Medicine, Karolinska Institutet, Karolinska University Hospital L8:03, SE-171 76 Stockholm, Sweden; 2 Department of Oncology-Pathology, Cancer Center Karolinska (CCK), Karolinska Institutet, Karolinska University Hospital R8:03, SE-171 76 Stockholm, Sweden

**Keywords:** peroxisome proliferator-activated receptor δ, microRNA-9, gene regulation, monocytes, macrophages, inflammatory response

## Abstract

PPARδ is involved in the inflammatory response and its expression is induced by cytokines, however, limited knowledge has been produced regarding its regulation. Since recent findings have shown that microRNAs, which are small non-coding RNAs that regulate gene expression, are involved in the immune response, we set out to investigate whether PPARδ can be regulated by microRNAs expressed in monocytes. Bioinformatic analysis identified a putative miR-9 target site within the 3′-UTR of PPARδ that was subsequently verified to be functional using reporter constructs. Primary human monocytes stimulated with LPS showed a downregulation of PPARδ and its target genes after 4 h while the expression of miR-9 was induced. Analysis of pro-inflammatory (M1) and anti-inflammatory (M2) macrophages showed that human PPARδ mRNA as well as miR-9 expression was higher in M1 compared to M2 macrophages. Furthermore, treatment with the PPARδ agonist, GW501516, induced the expression of PPARδ target genes in the pro-inflammatory M1 macrophages while no change was observed in the anti-inflammatory M2 macrophages. Taken together, these data suggest that PPARδ is regulated by miR-9 in monocytes and that activation of PPARδ may be of importance in M1 pro-inflammatory but not in M2 anti-inflammatory macrophages in humans.

## Introduction

Peroxisome proliferator-activated receptor δ (PPARδ) is a ubiquitously expressed member of the ligand-activated nuclear receptor superfamily. PPARδ has not only been shown to regulate genes involved in lipid and glucose homeostasis but also seems to play an important role in inflammation and innate immunity (1,2). Upon ligand binding, PPARδ is activated and releases the anti-inflammatory transcriptional suppressor protein B cell lymphoma-6 (BCL-6), which leads to suppression of inflammatory gene expression (3,4). Furthermore, activation of PPARδ has been shown to inhibit tumor necrosis factor α (TNFα)-induced nuclear factor of κ light polypeptide gene enhancer in B-cells 1 (NF-κB) activation (5). PPARδ has lately attracted attention for its involvement in different macrophage phenotypes in mice where it has been reported to induce a switch from pro-inflammatory M1 macrophages to the anti-inflammatory M2 phenotype in metabolically important organs such as liver and adipose tissue (6,7). The pro-inflammatory M1 macrophage phenotype is driven by Th1 cytokines e.g., interferon γ (IFNγ) and TNFα while the anti-inflammatory M2 macrophage phenotype is driven by Th2 cytokines e.g., interleukin 4 (IL-4) and IL-13. During atherosclerosis monocytes infiltrate the vessel wall, where these two subpopulations of macrophages can be detected (8).

Bone marrow-specific deletion of *Ppard* in mice renders adipose tissue and liver macrophages incapable of transition to the M2 phenotype, which in turn causes inflammation and metabolic derangement in adipocytes as well as hepatic dysfunction and systemic insulin resistance, respectively (6,7). These data demonstrate the importance of PPARδ for the inflammatory response and the dependence of PPARδ for maintaining the alternative macrophage phenotype in mice. In contrast, a recent human study investigating PPARδ activation during monocyte differentiation did not detect any increased expression of PPARδ in IL-4 induced M2 macrophages compared to untreated macrophages (9). While research on the function of PPARδ has mainly focussed on its activation, considerably less is known about the regulation of expression of human PPARδ. In the last few years microRNAs (miRNAs) have emerged as an important class of fine-tuning regulators of gene and protein expression. These transcripts are endogenous non-coding single-stranded RNAs ~22 nucleotides in length that bind to the 3′-untranslated region (3′-UTR) of their target mRNA and suppress expression either by inducing mRNA degradation or inhibiting protein translation (10). It is known that ~50% of all mammal protein-coding genes are directly regulated by miRNAs (11) and there are over two thousand known human miRNAs described today. MiRNAs are implicated in processes such as development and differentiation but many of them have also been reported to be aberrantly expressed in different forms of cancers. Computational studies have shown that recurrent networks exist consisting of specific transcription factors and specific miRNAs that both appear to regulate one another, thus coupling transcriptional and post-transcriptional regulation in order to create flexible expression (12). Identified miRNAs that have been shown to play key roles in monocytes and/or macrophages during inflammation include microRNA-9 (miR-9) and microRNA-155 (miR-155). MiR-9 is involved in the immune response by fine tuning the expression of a key member of the NF-κB family in monocytes and polymorphonuclear neutrophils (4) while the expression of miR-155 is increased during inflammation and has been implicated in macrophage polarization, where miR-155 modulates the switch between pro-inflammatory M1 and anti-inflammatory M2 phenotypes. MiR-155 directly inhibits the expression of IL-13 receptor α1 and thereby downregulates the anti-inflammatory IL-13 pathway in macrophages (13).

Of the human PPARs, both PPARα and PPARγ have been shown to be regulated by miRNAs (14–19), but until now no data are available regarding the regulation of PPARδ by miRNAs. Considering the important role that PPARδ plays in immunity, we explored the regulation of human PPARδ expression by miRNAs expressed in primary human monocytes during the inflammatory response and during macrophage polarization to M1 and M2 phenotypes, respectively.

## Materials and methods

### Bioinformatic sequence analysis

Prediction of miRNA binding to the PPARD 3′-UTR was performed by computer-aided algorithms obtained from TargetScan (http://www.targetscan.org), PicTar (http://pictar.mdc-berlin.de), miRanda http://www.microrna.org) and miRWalk (http://www.umm.uniheidelberg.de/apps/zmf/mirwalk). Approximately 3 kb of the miR-9 promoter was analyzed for putative PPREs using the MatInspector software (http://www.genomatix.de).

### Reporter gene constructs

Firefly luciferase reporter plasmids containing the 3′-UTR of the PPARδ gene and empty luciferase vector were obtained from Promega (Madison, WI, USA). The 36 nucleotides of PPARδ 3′-UTR containing the miR-9 binding site were cloned into psiCHECK-2 (Promega) using the restriction sites *Xho*I/*Not*I. The following oligonucleotides were utilized (the mutated sequences are shown in bold): miR-9WT-forw: 5′-tcgaTGTCTTCAGAGCAAAAGACTT GAGCCAT**CCAAAG**AA-3′; miR-9WT-rev: 5′-ggccTT**CTTT GG**ATGGCTCAAGTCTTTTGCTCTGAAGACA-3′; PPARδ-mir9Mut-forw: 5′-tcgaTGTCTTCAGAGCAAAAGACTTGA GCCAT**TAGCGT**AA-3′; PPARδ-mir9Mut-rev: 5′-ggccTT**AC GCTA**ATGGCTCAAGTCTTTTGCTCTGAAGACA-3′. The plasmids were sequenced and purified using the Endofree Plasmid Maxi kit (Qiagen, Düsseldorf, Germany).

### Cell culture and transfection

HEK293 cells were cultured in DMEM Glutamax (1 g/ml glucose, Invitrogen) containing 10% FBS, penicillin (100 U/ml) and streptomycin (100 μg/ml) at 37°C in 5% CO_2_. For luciferase assays, HEK293 cells were plated in 24-well tissue-culture dishes 24 h prior to transfection at a density of 80,000 cells per well. Cells were transfected with the luciferase reporters, 50 ng per well (Promega), together with pre-miR-9, 10 nM per well, or miRNA mimics negative control no. 1 (pre-miR-CON, Ambion, Foster City, CA, USA), 10 nM per well; 50 nM LNA-based anti-miR-9 (Exiqon, Vedbaek, Denmark) or 50 nM universal LNA-based negative control (anti-miR-CON) (Exiqon). All transfections were carried out in triplicates with Lipofectamine 2000 (Invitrogen, Carlsbad, CA, USA). Cells were lysed 24 h post-transfection using passive lysis buffer (Promega). Luciferase activity was determined using Dual Luciferase^®^ Reporter assay system (Promega) following the manufacturer’s instructions. Relative luciferase activity was determined by the ratio of renilla luciferase signal intensity to that of firefly luciferase for normalization.

Human monocytes were isolated from buffy coats as previously described (20). In brief, human peripheral blood mononuclear cells (PBMCs) were isolated from buffy coats by endotoxin-free Ficoll density gradient centrifugation. Monocytes were then separated from lymphocytes by high-density hyper-osmotic Percoll density gradient centrifugation and separated from platelets and dead cells on a low-density iso-osomotic Percoll density gradient. Monocytes were cultured in RPMI-1640 medium (Invitrogen) supplemented with penicillin-streptomycin, L-glutamine (2.05 mM) and 10% human AB-serum (Invitrogen). Monocytes were transfected with miRNA oligonucleotides as described above.

Monocytes were transfected with 50 nM anti-miR-9 or 50 nM anti-miR-CON (Exiqon) using Lipofectamine RNAiMAX (Invitrogen), following the manufacturer’s instructions. At 24 h after transfection, monocytes were stimulated with LPS (100 ng/ml) for 4 h.

Pro-inflammatory M1 and anti-inflammatory M2 macrophages were obtained by stimulating freshly isolated monocytes with recombinant human IFN-γ (20 ng/ml) and IL-4 (15 ng/ml) for 7 days, respectively. The human embryonic kidney cell line HEK293 was purchased from ATCC (Rockville, MD, USA). The cells were cultured in Dulbecco’s modified Eagle’s medium (1 g/ml glucose, Invitrogen) supplemented with 10% newborn calf serum, 100 U/ml penicillin and 100 μg/ml streptomycin (Invitrogen). All the cells were cultured at 37°C in 5% CO_2_. All cytokines were purchased from Peprotech (Rocky Hill, NJ, USA). GW501516 was synthesized by Synthelec AB, Sweden as described (21).

### RNA extraction, reverse transcription and quantitative real-time PCR

Total RNA was prepared using miRNeasy kit (Qiagen) according to the manufacturer’s instructions. The RNA concentration was determined by spectrophotometry. Total RNA (0.5 μg) was reverse transcribed (RT) into cDNA in a 20-μl reaction by a poly-dT primer using Superscript III™ (Invitrogen).

Quantification of miRNAs by TaqMan^®^ real-time PCR was carried out as described by the manufacturer (Applied Biosystems, Foster City, CA, USA). Briefly, 10 ng of template RNA was reverse transcribed using the TaqMan MicroRNA Reverse Transcription kit and miRNA-specific stem-loop primers (Applied Biosystems). RT product (1.5 μl) was introduced into 20-μl PCR reactions which were incubated in 96-well plates on the ABI PRISM^®^ 7900HT Sequence Detection System (Applied Biosystems) at 95°C for 10 min, followed by 40 cycles of 95°C for 15 sec and 60°C for 1 min. Target gene expression was normalized between different samples based on the values of U48 RNA expression.

The cDNA was amplified by real-time PCR as described (22). For the quantification, 15 ng of cDNA were amplified per reaction in the presence of TaqMan universal master mix (Applied Biosystems) and TaqMan Gene Expression Assays for PPARδ (Hs04187066_g1), PLIN2 (Hs00605340_m1), CPT1A (Hs00912676_m1), ANGPTL4 (Hs01101127_m1), TNFα (Hs01113624_g1), STAT1 (Hs01013996_m1), MRC1 (Hs00267207_m1) and STAT6 (Hs00598625_m1), all purchased from Applied Biosystems. Gene-specific PCR products were measured by means of the ABI PRISM^®^ 7900HT Sequence Detection System (Applied Biosystems). Target gene expression was normalized based on the values of the expression of cyclophilin A, PPIA (Hs04194521_s1) and 18s (Hs99999901_s1) obtained from Applied Biosystems.

### Statistical analysis

Wilcoxon rank-sum and signed-rank tests were employed to determine statistical differences between means of quantitative real-time PCR and luciferase assay data. P<0.05 was considered statistically significant.

## Results

### PPARδ is the direct target of miR-9

**Figure 1:**
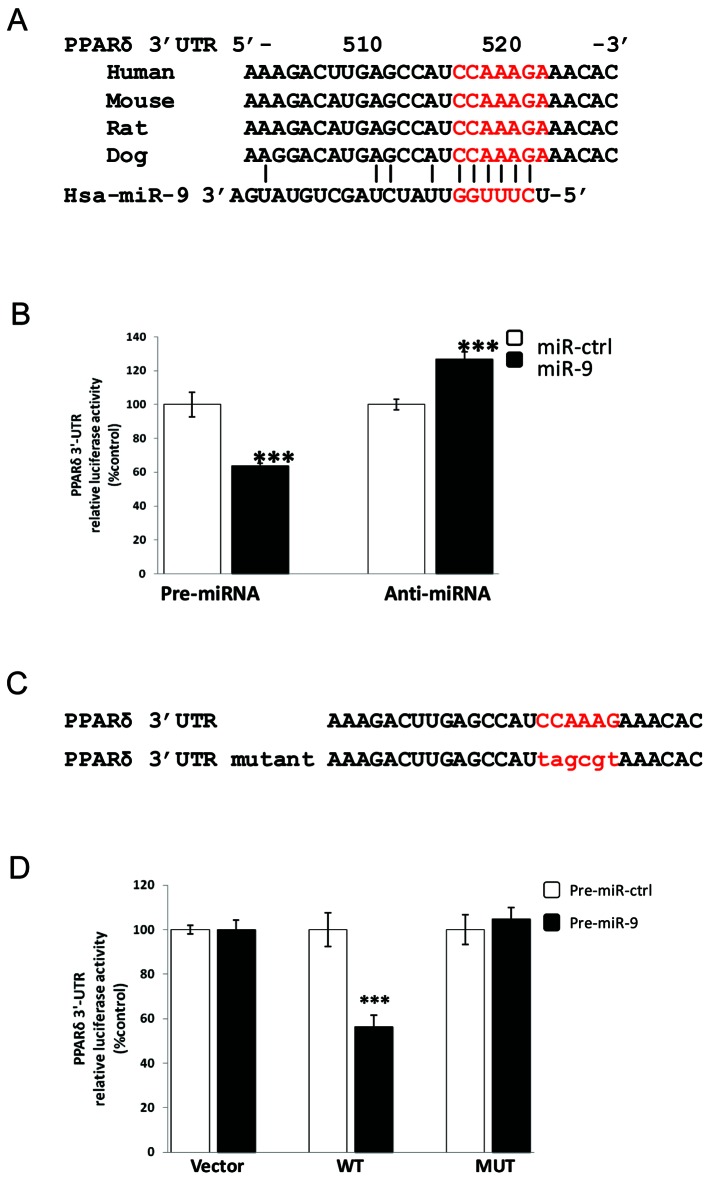
PPARδ is the direct target of miR-9. (A) DNA sequence alignment of the evolutionary conserved miR-9 binding site in the PPARδ 3′-UTR of mammals. The miR-9 seed match region is highlighted in red capital letters. (B) The relative expression obtained from reporter constructs containing the wild-type (WT) PPARδ 3′-UTR that were transfected into HEK293 cells together with pre-miR-9 or miR-9 inhibitor oligonucleotide (anti-miR-9). The luciferase activity was measured 24 h after transfection. (C) The nucleotides changed in the PPARδ 3′-UTR mutant construct are indicated in red lower case. (D) The relative expression obtained when HEK293 cells were transfected with reporter constructs containing wild-type (WT) or mutant (MUT) PPARδ 3′-UTR or empty renilla expression vector (vector) together with miR-9 precursor RNA (pre-miR-9) or miRNA precursor control (pre-miR-ctrl). The luciferase activity was measured 24 h after transfection. The experiments were repeated 3 times. A representative result is shown. Each bar represents the mean ± SD for 3 wells. ^***^P<0.001.

To investigate whether the expression of PPARδ in monocytes could be regulated by miRNAs during the inflammatory response and human macrophage polarization, bioinformatic analyses were performed. We screened the human PPARδ 3′-UTR for putative target sites of candidate miRNAs expressed in monocytes and/or macrophages (4,13,23). Four different computational prediction programs; TargetScan, PicTar, miRanda and miRWalk, were used. One putative miRNA target site for miR-9 was identified by all four programs. The identified target site for miR-9 in PPARδ 3′-UTR is highly conserved in many mammals including human, mouse, rat and dog (Fig. 1A).

In order to verify whether PPARδ is a direct target of miR-9, we performed 3′-UTR luciferase activity assays. Accordingly, 36 nucleotides encompassing the putative miR-9 binding site in the 3′-UTR of the PPARδ gene were cloned into a reporter plasmid containing the renilla luciferase gene. This reporter construct was transiently co-transfected with miR-9 mimic (pre-miR-9) or the specific inhibitor of miR-9 (anti-miR-9) as well as their respective control oligonucleotides into HEK293 cells and relative luciferase activities were determined 24 h after transfection (Fig. 1B). Overexpression of miR-9 reduced the luciferase activity down to 64% (P<0.001), whereas inhibition of endogenous miR-9 increased the luciferase activity up to 130% (P<0.001) compared with scrambled pre-miR or anti-miR control nucleotides, respectively (Fig. 1B), indicating that miR-9 directly targets PPARδ.

To examine whether the effects on transcription are mediated by the predicted miR-9 target site in the 3′-UTR of PPARδ, we changed 6 nucleotides within the miR-9 seed-matching sequence of the 3′-UTR of PPARδ to generate a construct named MUT (Fig. 1C). Mutation of the miR-9 seed-matching sequence led to a complete restoration of luciferase activity and reversed the inhibitory effect of miR-9 in the 3′-UTR of PPARδ (Fig. 1D), which shows that the effects are mediated through the identified miR-9 target site. Taken together, these results demonstrate that miR-9 directly regulates PPARδ expression by binding to the target site in the 3′-UTR of PPARδ mRNA.

### PPARδ mRNA expression is regulated by miR-9 in monocytes after LPS treatment

Since miR-9 has been shown to play an important role in the inflammatory response in monocytes, where it serves as a feedback controller of inflammation by suppressing NFκB1 signaling (4), the relevance of miR-9 regulation in relation to PPARδ expression was investigated in monocytes. To this end, primary monocytes were isolated from PBMC obtained from healthy donors followed by stimulation with LPS for different time periods. Expression of miR-9 and PPARδ was evaluated by qRT-PCR. To estimate the corresponding effects due to changes in human PPARδ protein expression, we analysed PPARδ target genes, such as perilipin-2 (*PLIN2*), carnitine palmitoyltransferase 1α (*CPT1A*) and angiopoietin-related protein 4 (*ANGPTL4*), none of which had putative miR-9 target sites as evaluated by bioinformatic analyses (24).

**Figure 2:**
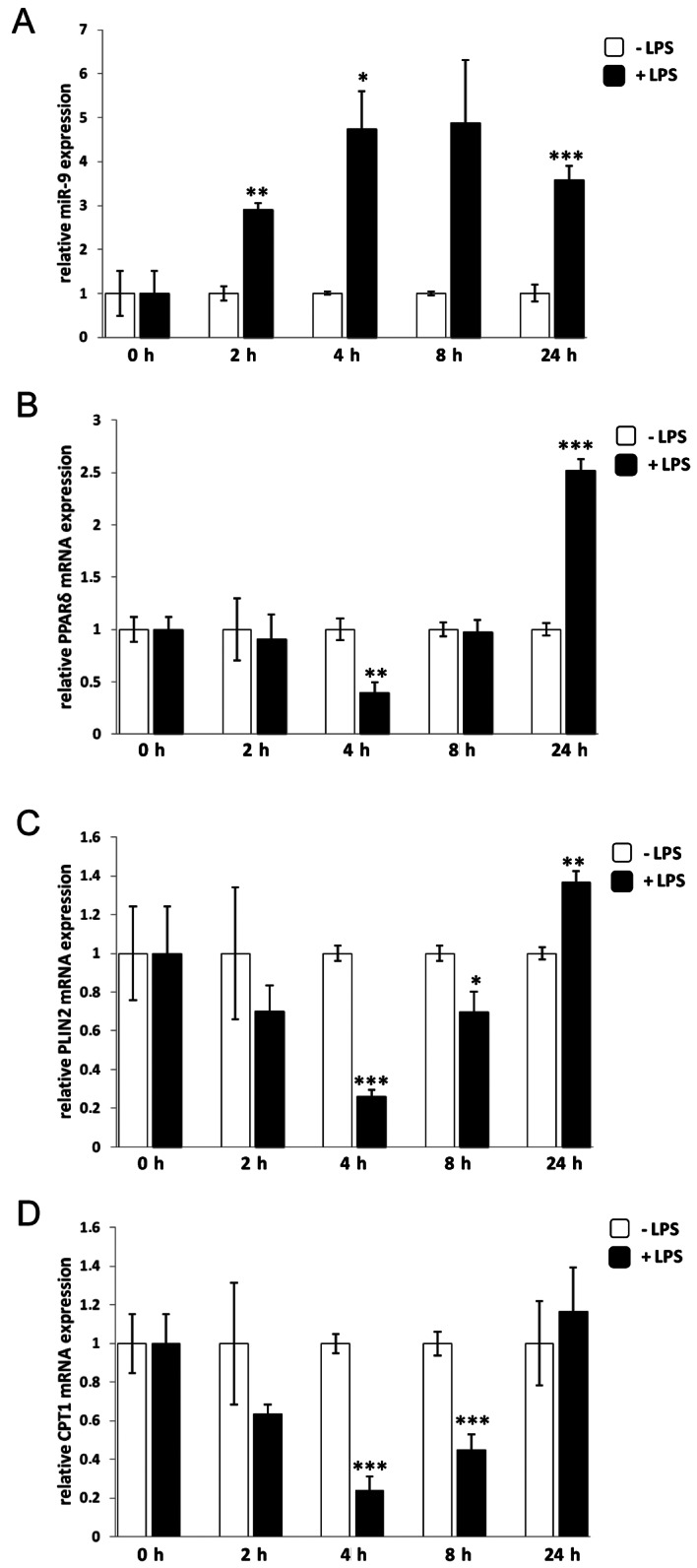
PPARδ mRNA expression is suppressed by miR-9 in monocytes after LPS treatment. Human primary monocytes were seeded in 6-well plates (2×10^6^ cells/well) and treated with LPS (100 ng/ml) for 2, 4, 8 and 24 h. The expression of (A) miR-9, (B) PPARδ, (C) PLIN2 and (D) CPT1A was then measured by real-time PCR. The experiment was repeated 3 times. A representative result is shown. Mir-9 expression was normalized to U48 expression while PPIA was used for normalization of PPARδ, PLIN2 and CPT1 expression. Each bar represents the mean ± SD for 3 wells. ^*^P<0.05, ^**^P<0.01, ^***^P<0.001.

In line with the findings by Bazzoni *et al*(4), miR-9 levels increased rapidly after 2 h and remained increased until 24 h after treatment with LPS (Fig. 2A). The PPARδ mRNA level, on the other hand, was unchanged until 4 h after LPS treatment when it was suppressed by 60% compared to control cells treated with vehicle only. The suppression was abolished after 8 h and increased at 24 h of LPS stimulation (Fig. 2B). Analysis of the PPARδ target genes PLIN2 and CPT1A showed 80 and 76%, respectively, reduced expression levels compared to unstimulated control cells after 4 h of LPS treatment and this reduction remained until 8 h, followed by an increase at 24 h (Fig. 2C and D). Analysis of another PPARδ target gene, ANGPTL4, showed the same trend as PLIN2 and CPT1A (data not shown). Thus, *PLIN2* was chosen as the PPARδ target gene in the following analyses due to its relatively high abundance. Taken together, the results suggest a model in which upregulation of miR-9 upon LPS stimulation in monocytes results in a decrease in PPARδ expression thereby suppressing its corresponding target genes at an early time-point (4–8 h), indicating that PPARδ is regulated by miR-9 in monocytes after LPS treatment.

### Suppression of miR-9 upregulates the mRNA expression of PPARδ and its target gene PLIN2 in human primary monocytes

**Figure 3:**
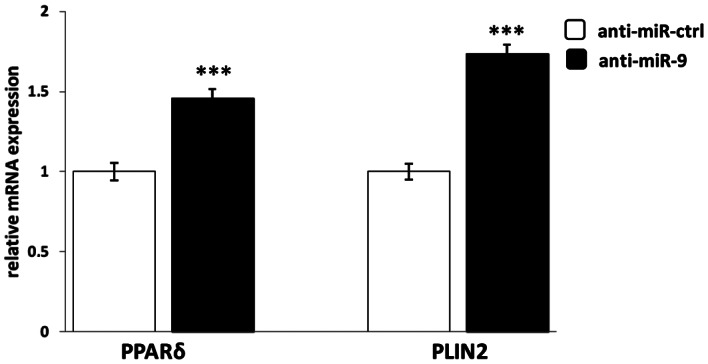
Suppression of miR-9 upregulates the mRNA expression of PPARδ and its target gene PLIN2. Human primary monocytes were transfected with anti-miR-9 or miRNA inhibitor control (anti-miR-ctrl) for 24 h, followed by LPS treatment for 4 h. The expression of PPARδ and its target gene PLIN2 was then examined by real-time PCR. The experiment was repeated 3 times. A representative result is shown. Each bar represents the mean ± SD for 3 wells. ^***^P<0.001.

The influence of miR-9 on PPARδ expression in monocytes during the inflammatory response is unknown. Thus, we further evaluated regulation by miR-9 of PPARδ expression in human primary monocytes stimulated with the pro-inflammatory agent LPS. Human primary monocytes were transfected with the specific anti-miR-9 or its control oligonucleotides for 24 h, followed by LPS stimulation for 4 h. In order to measure the effect of miR-9 the mRNA expression of PPARδ and its target gene PLIN2 was quantified by qRT-PCR. As shown in Fig. 3, specific inhibition of miR-9 significantly increased PPARδ and PLIN2 mRNA expression 40 and 70%, respectively, compared to scrambled control oligonucleotides (anti-miR-ctrl). This result suggests that miR-9 downregulates the expression of PPARδ and its target gene PLIN2 and further confirms the regulatory link between PPARδ and miR-9.

### Both PPARδ mRNA and miR-9 expression are higher in M1 than in M2 macrophages

**Figure 4:**
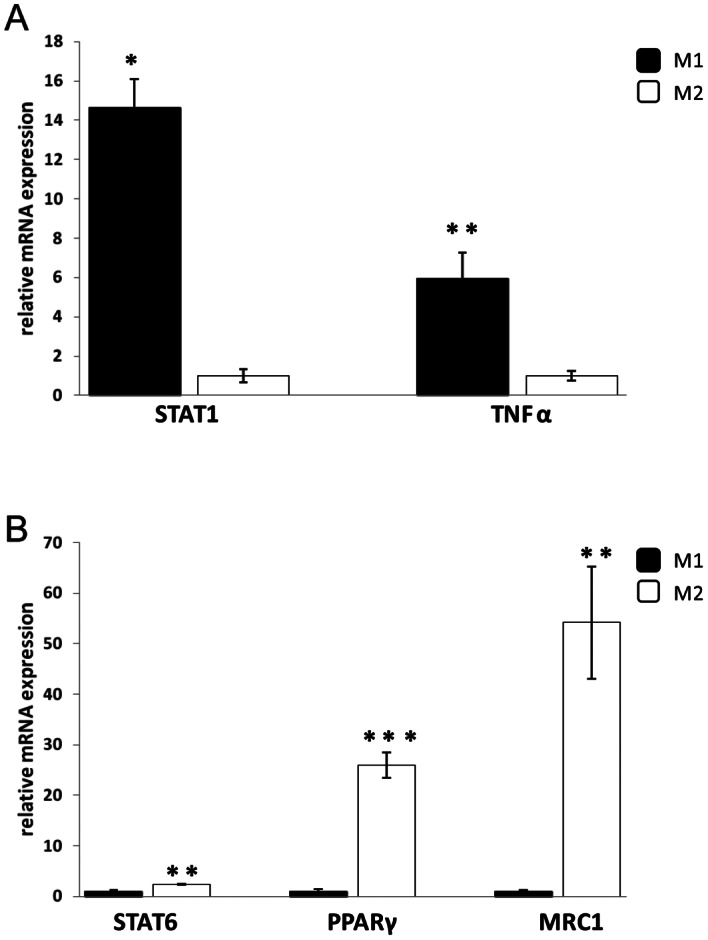
Expression of markers for M1 and M2 macrophages. Human primary monocytes were seeded in 6-well plates (2×10^6^ cells/well). M1 and alternative M2 macrophages were obtained by stimulating the monocytes with recombinant human IFN-γ (20 ng/ml) and IL-4 (15 ng/ml) for 7 days, respectively. The cells were harvested and preceded to quantitative real-time PCR for measurement of (A) M1 (STAT1 and TNFα) and (B) M2 (STAT6, PPARγ and MRC1) markers. The experiment was repeated 4 times. A representative result is shown. STAT1, TNFα, STAT6, PPARγ and MRC1 expression were normalized to 18s expression. Each bar represents the mean ± SD for 3 wells. ^*^P<0.05, ^**^P<0.01, ^***^P<0.001.

PPARδ has been suggested to play a role in the switch from pro-inflammatory M1 phenotype to anti-inflammatory M2 phenotype in macrophages. To study whether miR-9 expression was involved in the polarization of M1 and M2 macrophage phenotypes through modulation of PPARδ expression, human primary monocytes were differentiated into M1 and M2 macrophages, respectively. Hence, human primary monocytes were cultured in the presence of either IFNγ to promote a shift to pro-inflammatory M1 macrophages, or IL-4 to polarize the macrophages into the anti-inflammatory M2 phenotype. As expected the M2 marker, mannose receptor C type 1 (MRC1), showed higher expression in M2 compared with M1 or untreated macrophages using FACS analysis (data not shown). TaqMan analysis revealed that the expression of TNFα and signal transducer and activator of transcription 1 (STAT1), which are markers of M1, were higher in M1 compared to M2 macrophages while the mRNA expression of MRC1 and STAT6 were higher in M2 compared to M1 macrophages (Fig. 4). In addition, PPARγ mRNA was also analysed since its expression has been shown to correlate with markers for M2 macrophages. As shown in Fig. 4B, PPARγ mRNA levels were significantly increased in M2 compared with M1 macrophages. These results demonstrate that two distinct macrophage phenotypes, M1 and M2, were obtained.

**Figure 5:**
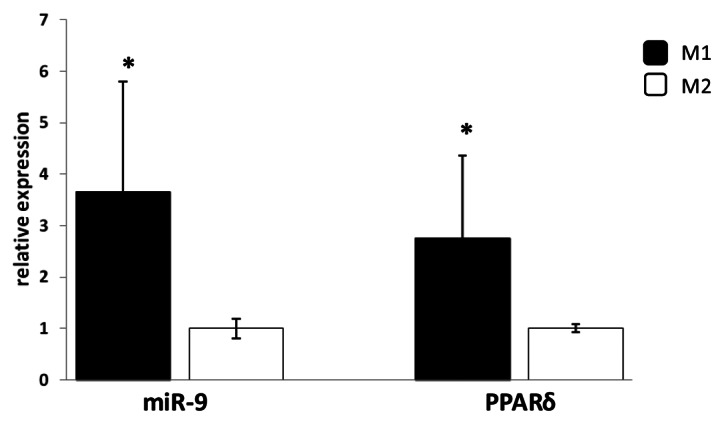
Both the expression of PPARδ mRNA and miR-9 are higher in M1 than in M2 macrophages. The expression of miR-9 and PPARδ mRNA was measured in macrophages, after 7 days of treatment with IFN-γ or IL-4 to obtain the M1 and M2 phenotypes, respectively. The experiment was repeated 5 times using monocytes from 6 different donors. Mir-9 expression was normalized to U48 expression and PPARδ expression was normalized to 18s expression. Means ± SD of 5–6 independent experiments are shown. ^*^P<0.05.

Analyses of the expression of PPARδ mRNA and miR-9 showed that their levels are significantly increased in M1 compared with M2 macrophages (Fig. 5), which suggest that PPARδ and miR-9 might be of importance in modulating the pro-inflammatory M1 human macrophage phenotype.

### Expression of miR-9 in relation to PPARδ agonist treatment

The polarization towards the M1 phenotype is important for macrophages exposed to pro-inflammatory stress. To further investigate the interplay between PPARδ and miR-9 in monocytes and macrophages during the inflammatory response, we examined whether miR-9 can be regulated by PPARδ. Since pri-mir-9-1 is the only primary miR-9 transcript induced by LPS (4), we set out to analyse whether a PPAR response element (PPRE) exists in the pri-mir-9-1 promoter region. *In silico* analysis of the promoter region of pri-mir-9-1 revealed five putative PPREs within 2.5-kb upstream of the transcriptional start site (data not shown).

**Figure 6:**
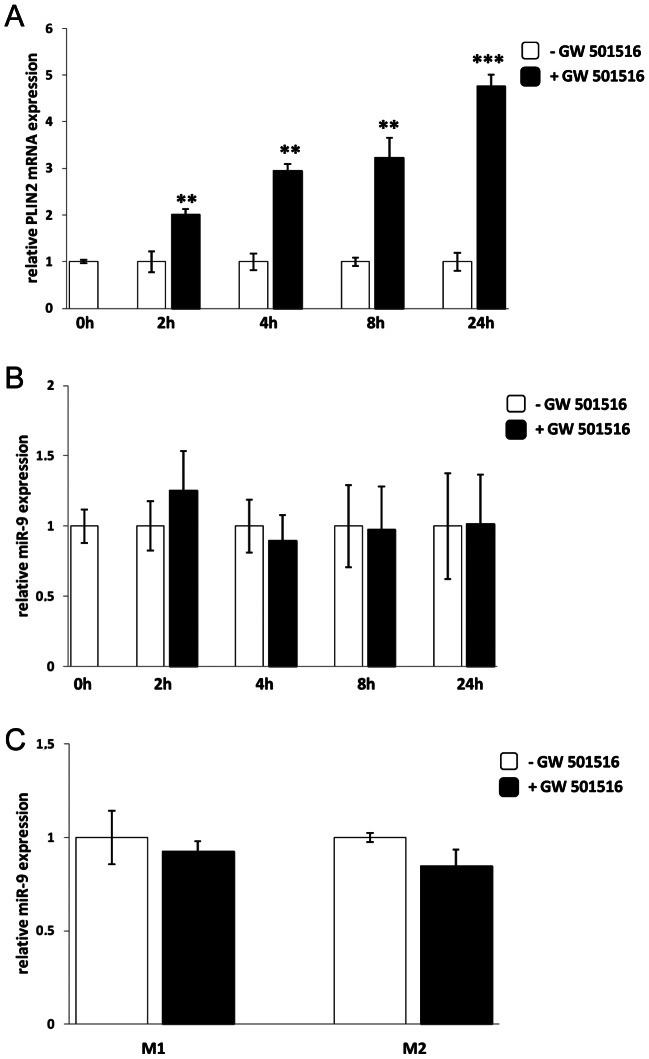
The miR-9 expression is not regulated by PPARδ in monocytes. (A) Human primary monocytes were seeded in 6-well plates (2×10^6^ cells/well) and treated with 100 nM PPARδ agonist (GW501516) for 2 h followed by isolation of RNA from the cells. To verify the potency of GW501516, mRNA expression of the PPARδ target gene, PLIN2, was analysed by real-time PCR. (B) The expression of miR-9 is shown from the same cells. (C) The expression of miR-9 in M1 and M2 macrophages, respectively, is shown after 4 h of treatment with GW501516. The experiment was repeated in monocytes from two individuals and in macrophages from two individuals. Representative results from these experiments are shown. White bars show results from no GW501516 treated cells and the results were set to one. PLIN2 expression was normalized to PPIA expression and miR-9 expression was normalized to U48 expression. Each bar represents the mean ± SD for 3 wells. ^*^P<0.05.

Next, we explored whether PPARδ activation could induce miR-9 expression in human monocytes and/or macrophages using the specific PPARδ agonist, GW501516. Human monocytes were isolated from PBMC and stimulated with GW501516 for 2, 4, 8 and 24 h. Since PLIN2 gene expression is regulated by PPARδ, PLIN2 mRNA levels were measured at the different time-points to serve as a positive control and to ensure that GW501516 had activated PPARδ. As expected PLIN2 mRNA expression was upregulated at the different time-points analysed after adding GW501516 (Fig. 6A). However, the miR-9 expression was not induced at any of the different time-points of treatment with GW501516 (Fig. 6B), nor was there any difference of miR-9 expression in M1 or M2 macrophages after GW501516 treatment (Fig. 6C).

### PPARδ agonist treatment results in upregulation of PPARδ target genes in M1 but not in M2 macrophages

**Figure 7:**
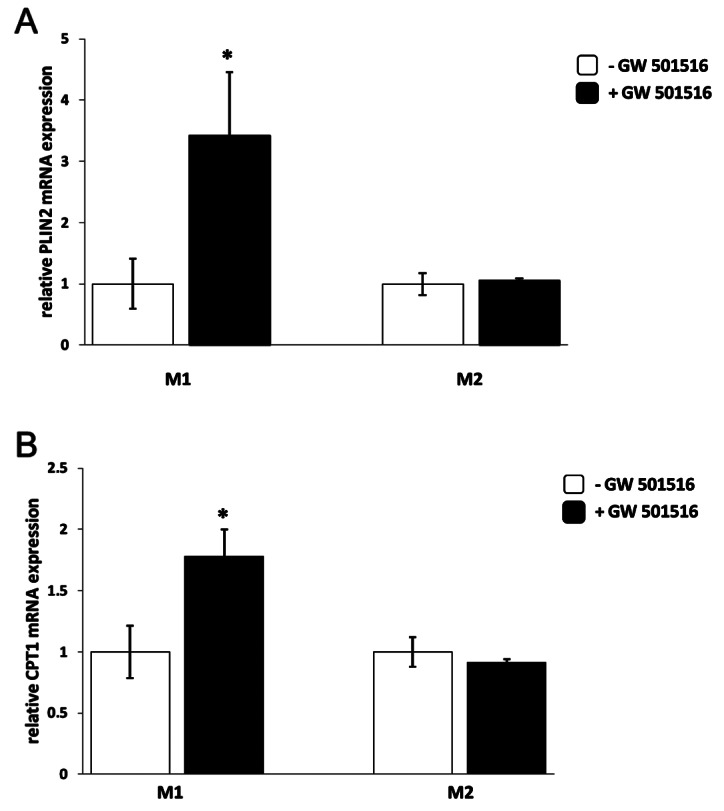
GW501516 induces PLIN2 mRNA expression in M1 but not in M2 macrophages. Pro-inflammatory M1 or anti-inflammatory M2 macrophages were obtained after 7 days of stimulation with IFN-γ or IL-4, respectively, followed by treatment with the PPARδ agonist, GW501516, for 4 h. The relative mRNA expression of the PPARδ target genes, (A) PLIN2 and (B) CPT1A, was analyzed in the two phenotypes of macrophages. A representative result is shown. PLIN2 and CPT1 expression were normalized to 18s expression. Each bar represents the mean ± SD for 3 wells. ^*^P<0.05.

In order to evaluate the influence of PPARδ on the expression of its target genes, M1 and M2 macrophages were subjected to GW501516 treatment for 4 h. Human primary monocytes that were differentiated into macrophages of the pro-inflammatory M1 phenotype responded to the treatment with GW501516 by upregulation of PLIN2 and CPT1A mRNA while macrophages differentiated into the anti-inflammatory M2 phenotype did not (Fig. 7). Another PPARδ target gene that was analysed, ANGPTL4, also showed induction of mRNA expression upon GW501516 treatment only in M1 but not in M2 macrophages (data not shown).

## Discussion

It has been suggested that the primary regulation of the human PPARδ gene is not at the transcriptional level. Instead, PPARδ is known to be regulated at the post-translational level by the presence of ligands along with specific cofactors that are of major importance for PPARδ activation and function. In this study, we show that PPARδ is also regulated at the post-transriptional level by miR-9. Upregulation of miR-9 results in the direct repression of PPARδ, the mRNA levels of which are found to be higher in pro-inflammatory M1 than in anti-inflammatory M2 macrophages.

MiR-9 has been shown to be of importance during the immune response (4), post-traumatic stress (25), neuronal differentiation (26), different forms of cancers (27,28) and exocytosis of insulin from pancreatic islets (29). Here, we show that miR-9 is upregulated in human monocytes after LPS treatment while PPARδ and its target genes PLIN2, CPT1A and ANGPTL4 are downregulated 4–8 h after LPS-treatment compared with untreated cells. The suppression, intriguingly, was abolished after 8–24 h of LPS stimulation despite the presence of continued high levels of miR-9. One possible explanation might be that the effect and activity of miR-9 are influenced due to interactions with RNA-binding proteins (30). Another reason might be that LPS triggers other factors that in turn could affect the expression of PPARδ. Indeed, Tan *et al*(31) have shown that pro-inflammatory cytokines, such as TNFα, can both increase PPARδ expression via the stress kinase signaling pathway and trigger the production of ligands for this receptor, which is in agreement with our findings that PPARδ is upregulated after 24 h of LPS treatment. The inhibition of PPARδ by miR-9 at the early time-points might be a mechanism to delay the effect of PPARδ action early in inflammation to prevent PPARδ from suppression of NF-κB since NF-κB and PPARδ have been shown to be able to crosstalk and inhibit the function of each other (32). The inhibitory effect by miR-9 on PPARδ expression was confirmed by transfection of anti-miR-9 that sequesters mature miR-9 thus inhibiting its biologic function into monocytes, which resulted in induction of PPARδ and PLIN2 mRNA levels.

Hence, the miR-9 mediated inhibition of PPARδ expression in monocytes may constitute a negative feedback loop, modulating the levels of the receptor during inflammation. This finding regarding miR-9 expression in monocytes is in agreement with a previous study, which showed that the expression of miR-9 is dramatically increased after treatment with LPS (4). Bazzoni *et al* showed that the miR-9 targeting of NF-κB at the mRNA level constitutes a feedback loop of the inflammatory response. Other studies have confirmed the regulatory function of miR-9 on the mRNA of NF-κB in both ovarian and gastric cancers where miR-9 has been shown to act as a tumor suppressor (33,34). Of note, in the monocytic cell line THP1, miR-9 is abundantly expressed and there is no induction of miR-9 upon LPS stimulation (unpublished data), therefore all the current experiments were carried out in human primary monocytes or macrophages.

Here, the regulation of PPARδ by miR-9 was examined in relation to the pro-inflammatory M1 and anti-inflammatory M2 macrophage phenotypes. The role of PPARδ in the pro-inflammatory M1 macrophages has not been investigated before while PPARδ has been suggested to be of importance in macrophages with the anti-inflammatory M2 phenotype, both in adipose tissue and liver of mice (6,7) although this seems not to be the case in humans (9).

Monocytes were differentiated to macrophages of the M1 and M2 phenotypes by stimulation with IFNγ and IL-4, respectively, for 7 days. To confirm this, several markers that are commonly used to distinguish between the two distinct M1 and M2 macrophage phenotypes were analyzed. In agreement with other reports the expression of STAT1 and TNFα were higher in M1 than M2 macrophages while STAT6, mannose receptor type C and PPARγ were higher in M2 than in M1 macrophages (35).

In this study, both the expression of PPARδ and miR-9 were higher in pro-inflammatory M1 than in anti-inflammatory M2 macrophages, indicating the potential involvement of PPARδ and miR-9 in modulating the M1 macrophage phenotype. As expected TNFα expression was higher in M1 than in M2 macrophages and the PPARδ ligand, GW501516, did not influence the expression of miR-9 in M1 macrophages. These results indicate that the pro-inflammatory cytokines are responsible for the induction of both miR-9 and PPARδ expression, which is in agreement with previous studies (4,36,37). In addition, the expression of PPARδ target genes was induced by GW501516 in macrophages of the M1, but not the M2 phenotype, which probably is due to the fact that PPARδ is mainly expressed in M1 macrophages while PPARγ is predominantly expressed in M2 macrophages in humans (6–9).

Our data show the regulation of PPARδ by miR-9 in monocytes. Whether this regulation might play a role in foam cell formation and initiation of atherosclerosis remains to be elucidated. However, activation of PPARδ does not seem to promote the polarization into M2 macrophages (unpublished data). In contrast to the studies by Kang *et al* and Odegaard *et al*(6,7), our study of human macrophages showed a higher PPARδ mRNA expression in M1 macrophages compared to M2 macrophages. This result supports the data from Bouhlel *et al*(9), who showed that PPARδ expression was lower in M2 macrophages compared to resting (untreated) macrophages. These data highlight differences between human and mouse macrophages and suggest that they may be regulated by different factors.

The importance of miR-9 in monocytes and macrophages as a response to proatherogenic factors is further demonstrated by a recent report showing that miR-9 is significantly upregulated in monocytes and macrophages after exposure to oxLDL (38). One purpose of the inhibition of PPARδ by miR-9 in monocytes could be to induce a block in monocyte expansion. Whether PPARδ inhibits or stimulates cell proliferation is still debated (39), but during the inflammatory response cells must focus on repair and disposal of causal pathogens rather than undergoing cell divisions. In this context, PPARδ has been shown to function as a sensor of apoptotic cells in macrophages by promoting the clearance of apoptotic cells and suppressing autoreactive immune responses (40).

Importantly, a miRNA does not only target one gene, but frequently rather modestly regulates a number of genes belonging to the same network. The long list of putative miR-9 targets identified by computational prediction programs contains genes working in a close network with PPARδ, for example PPARα, RXRα, PGC1α, FOXO and BCL-6. If all these genes are miR-9 regulated, the outcome of miR-9 regulation would be due to effects on the whole network rather than only to PPARδ. Putative target genes of PPARδ can also be found on the miR-9 target list, such as ABCA1, ABCD1, GOT1 and PDK4. The effects of targeting these genes would cause similar results as targeting of PPARδ itself, but resulting in a larger net effect. To draw firm conclusions, however, a functional approach for each gene would be required in order to validate whether these genes are true miR-9 targets, which although relevant, was outside the scope of this study.

In conclusion, we have identified miR-9 as a regulator of PPARδ expression in monocytes through direct targeting of a specific sequence with the 3′-UTR of PPARδ. These data show that miR-9 and PPARδ are involved in central signalling pathways during the inflammatory response in monocytes. Interestingly, activation of PPARδ by GW501516 showed that the PPARδ target genes expressed in the pro-inflammatory M1 macrophages were induced while the expression of the same genes were unaffected in the anti-inflammatory M2 macrophages, which may suggest that PPARδ is of importance in M1 pro-inflammatory macrophages.
